# Dual-Action Costs and Benefits in a Uni-Modal Single-Onset Paradigm

**DOI:** 10.1027/1618-3169/a000604

**Published:** 2024-04-11

**Authors:** Tim Raettig, Lynn Huestegge

**Affiliations:** ^1^ Department of Psychology (III) - Psychological Methods, Cognition, and Applied Research, Institute of Psychology, Julius-Maximilians-Universität of Würzburg, Germany

**Keywords:** multiple-action control, dual-action costs, dual-action benefits, action planning, inhibition

## Abstract

**Abstract:** While performing two actions at the same time has mostly been associated with reduced performance, several recent studies have observed the *opposite* effect, that is, dual-action *benefits*. Previous evidence suggests that dual-action benefits result from single-action inhibitory costs – more specifically, it appears that under certain circumstances, single-action representations are derived from dual-action representations by removing (i.e., inhibiting) one of the component actions. In the present paper, we investigated if this is tied to the presence of multi-modal response demands (i.e., responses making use of two different effector systems). We implemented a very simple experimental paradigm where participants responded to a single stimulus with zero, one, or two *uni*-modal responses. As predicted, we did not observe dual-action benefits, but rather significant dual-action costs. Furthermore, a trial-by-trial sequence analysis revealed that alternations between both single-action responses were associated with significantly better performance than all other types of action switches. This can be accounted for by assuming that actions are represented as “feature bundles” and that switching a single, *binary* distinctive feature of an action to its *opposite* is relatively easy.


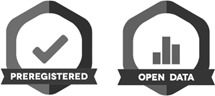


Performing two actions at the same time is often associated with reduced performance, that is, higher error rates (ERs) and slower reaction times (RTs; Fagot & Pashler, 1992; Navon & Miller, 1987; Pashler, 1994). Various mechanisms have been proposed to explain such dual-action costs (DACs), for example, a bottleneck during response selection (Pashler, 1994), limited cognitive capacity that is shared between action-control processes (Meyer & Kieras, 1997; Tombu & Jolicoeur, 2003; Wickens, 2008), or crosstalk between concurrent or sequential task demands (Huestegge, 2011; Navon & Miller, 1987). However, we have recently observed the *opposite* effect in a number of studies where performance *improved* when participants had to execute two actions at the same time (dual-action benefits, DABs; Huestegge & Koch, 2014; Kürten et al., 2022; Raettig & Huestegge, 2018, 2021, 2023).

It has been argued elsewhere that DABs result from action inhibition – more specifically, deriving a single-action representation from a dual-action representation by removing one of the component actions (Huestegge & Koch, 2014; Raettig & Huestegge, 2018, 2021, 2023). The underlying assumption here is that under certain circumstances, single actions are actually cognitively represented in terms of what *not* to do (“inhibitory coding,” cf. the Improved Inhibitory Coding Model, IICM; Raettig & Huestegge, 2021), relative to a prepotent dual-action default. Inhibitory coding stands in contrast to “executive coding” (the – often implicit – standard assumption of most theories of action control), that is, the specification of actions in terms of what to *do*.

To illustrate, the simplest cognitive representation of a single action would be something akin to “execute action A.” However, in a context where action A often has to be executed at the same time as a different action B, it may be efficient to execute *both* actions by default. Single actions can then be represented subtractively (e.g., “*inhibit* action B” resulting in the *execution* of action A). Assuming that executing the default is automatized – allowing for dual actions to be simply represented as “inhibit nothing” – DABs can then be explained parsimoniously in this inhibitory-coding framework (in essence: “inhibit nothing” – resulting in dual-action execution – is less costly than “inhibit something” – resulting in single-action execution). When actions are coded *executively*, on the other hand, DACs result since executive dual-action representations (“execute action A” + “execute action B”) are more complex than executive single-action representations (“execute action A”).^1^

Crucially, the IICM conceptualizes cognitive coding as highly *flexible* (“representational flexibility theory”; see Huestegge & Strobach, 2021), meaning that actions are only taken to be coded inhibitorily if that is the most efficient way of action representation in a given context. Generally speaking, inhibitory coding is more efficient than executive coding since it allows for simpler, noncompositional cognitive representations of dual actions (“inhibit nothing” in contrast to “execute A + execute B”) while single-action representations are equally complex for both coding schemes (“inhibit A,” “inhibit B” and “execute A,” “execute B,” respectively), meaning that in sum, inhibitory action representations are less resource demanding. However, DABs have as-of-yet only been observed in a relatively small number of studies – which is surprising given its theoretical superiority in terms of cognitive resource use.

Importantly, our own experiments always made use of two response modalities (i.e., two effector systems), manual versus oculomotor (Huestegge & Koch, 2014; Kürten et al., 2022) and manual versus vocal (Raettig & Huestegge, 2018, 2021, 2023), respectively. Thus, there is correlational evidence suggesting that multi-modality may be a critical precondition for the occurrence of DABs, and there is a plausible causal mechanism, too: DABs are (at least partly) driven by differential inhibitory costs when single actions are coded inhibitorily, and inhibitory coding may only happen when the context allows for easy, cost-efficient action inhibition. This, in turn, may rely on clearly separable “inhibition targets” with as little potential for interference or crosstalk as possible (Kürten et al., 2023; Paas Oliveros et al., 2023; Schacherer & Hazeltine, 2021; Schuch & Koch, 2004).

For example, stopping all *manual* actions while at the same time still executing a right *vocal* action (in a multi-modal response context) may be considerably faster and less error-prone than *stopping* a *left* manual action while at the same time still *executing* a *right* manual action (in a uni-modal response context). In a multi-modal setting, inhibitory coding of single actions (e.g., “inhibit the manual action” to execute the vocal action) relative to a highly automatized and – as a result – *low-cost* dual-action default would then result in (1) overall reduced representational complexity (equivalent complexity for single actions, lower complexity for dual actions) and (2) as a direct consequence, DABs. In a uni-modal setting, on the other hand, inhibitory coding of single actions would come with an additional “crosstalk penalty,” with the increase in *processing* costs (implementing difficult inhibition in single-action trials) more than offsetting the reduction in *representational* costs (simplified – in particular, noncompositional – dual-action coding), leaving executive coding (with the ensuing DACs) as the better option.

In the present study, we tested the hypothesis that *uni*-modal single and dual actions are not conducive to inhibitory coding (Hypothesis 1). To this end, we developed a novel paradigm utilizing a uni-modal single-onset procedure. We predicted that we would observe dual-action *costs*, not *benefits*, in this setting (Prediction 1) – in contrast to previous results from experiments with *multi*-modal responses. As a secondary aim, we wanted to take a detailed look at trial-by-trial sequence effects, which may be a particularly important source of DABs in the single-onset paradigm. It has been shown that RT DABs are specifically tied to action switches (Raettig & Huestegge, 2021) – for example, *inhibiting* an unwarranted vocal response in a single-manual trial is harder when the previous trial required the *execution* of a vocal response (i.e., trial *n* − 1 = single-vocal or dual) than when the previous trial also required vocal inhibition (i.e., trial *n* − 1 = single-manual), resulting in higher inhibitory costs in single-action switch trials. Thus, if – contrary to Hypothesis 1 – there were even weak inhibition-based DABs in uni-modal settings, switch trials would be where we would expect to see them.

Analyzing sequence effects is interesting for another reason, too: So far, we have only considered different “reference frameworks” of action representation (i.e., executive coding/“what to *do*” vs. inhibitory coding/“what *not* to do”) – but how exactly is the *what* coded? Based on the work by Rosenbaum (1980), it has been suggested that actions may be cognitively represented as specifications of “distinctive features” (also see Chomsky, 1965; Jakobson & Halle, 1956; and Frings et al., 2020, although we are only talking about action features here, not about binding action features to stimulus features and/or action effects). For example, in the present experiment, pressing the left button could be represented as a specification of three such features, effector (“index finger”), movement (“down”), and laterality (“left”). Left-to-right and right-to-left switches (from trial to trial) would then simply require inverting the polarity of the “laterality” feature, which is likely comparatively easy (if it is not left, it is right, and vice versa). In contrast, changing the number of actions that have to be executed (e.g., switching from a single- to a dual-action trial) cannot be achieved by a mere feature switch within the same base action but instead requires a fundamental modification of the previous response configuration. Thus, if actions are coded as bundles of distinctive features as described above (Hypothesis 2), directional switches would be the only kind of switch not requiring adding or removing actions and should thus be associated with better performance than all other kinds of switches (Prediction 2).

## Methods

### Participants

A priori power analyses were conducted using a simulation approach based on the R package Superpower (Lakens & Caldwell, 2021). Sample size was determined from previous research (Raettig & Huestegge, 2021). Based on the lowest observed ANOVA effect size in this previous paper, $\eta_{p}^{2}$ = 0.31 for the ER main effect of response condition in the vocal modality, we conservatively computed a minimum sample size of 26 to achieve a power of 1−β=0.95 at α=0.001. The script used to arrive at this value is available from the OSF repository (see below).

Twenty-seven university students with normal or corrected-to-normal vision participated in the experiment (16 males, *M*_age_ = 26.4 years, *SD* = 6.1, range = 22–37). All participants were native speakers of German and gave informed consent before completing the study.

### Apparatus, Stimuli, and Procedure

The experiment was run on a desktop computer with the Windows 10 operating system, using PsychoPy 1.83.04. A 19-inch thin-film transistor screen (1280×1024 pixels resolution) was used for stimulus presentation. We recorded responses with a USB keyboard. Participants used the index fingers of the left (“d” key) and right (“k” key) hands to respond. Stimuli (colored circles with a 400-pixel diameter) were presented centrally on a black background.

After reading instructions presented on the computer screen, participants performed a 36-trial training session. The experiment was made up of 360 trials (90 per action condition) with conditions being presented in a randomized fashion without blocking. A white central fixation cross marked the beginning of each trial.^2^ After 500 ms, a colored circle (the signal) was presented for 500 ms. The color of the signal (red, yellow, green, or blue) indicated the response condition (Left: “press the ‘d’ key”; Right: “press the ‘k’ key”; Dual: “press both the ‘d’ key and the ‘k’ key”; Null: “do not press any key”, independent variable: action). Participants were told to respond as quickly and as accurately as possible once the signal was presented. There were no explicit instructions regarding action sequencing in the dual-action condition. Responses occurring later than 650 ms post signal onset were recorded as too late. 500 ms after the stimulus had disappeared, participants received visual feedback if their response was either wrong or not made in time. This feedback was presented for 1,000 ms (if the response had been correct, a black screen was shown instead).

Furthermore, every four trials, participants were visually instructed to (1) “think of [X],” (2) “not think of [X],” or (3) “think about anything” (independent variable: mode), with X being one of 10 simple thought objects (e.g., “a pink elephant”). Each thought object was presented in both Modes 1 and 2, and all three modes were equally frequent. The mode instruction was presented before the fixation cross appeared. Participants were instructed to keep with their current mode/thought assignment until they got a new one (i.e., each thought/mode combination was valid for four trials). This was an exploratory manipulation that did not have any replicable effects and was immaterial to our main research question; consequently, we do not discuss it any further. However, we have included a statistical analysis in ESM 2.

### Data Analysis

We mainly analyzed ERs and RTs as a function of the within-subject independent variables current action (action on trial *n*) and previous action (action on trial *n* − 1). Since the intermittent mode instructions on every fourth trial intervened between the previous and current actions, we only analyzed instruction-free trials here. As per Prediction 1, we expected a main effect of current action due to worse performance for dual actions (action = Dual) than for single actions (action = Left or action = Right). Such DACs would lend support to Hypothesis 1 (no inhibitory coding – and thus, no inhibition-based DABs – in uni-modal settings). Furthermore, we expected an interaction between current action and previous action for two reasons: (a) Prediction 2, that is, directional switches should be associated with better performance than all other kinds of switches (lending support to Hypothesis 2: directional switches do not require adding or removing actions, but a simple feature switch); and (b) general action-repetition benefits (based on an extensive literature showing that repeating actions is easier than switching actions; see, e.g., Bertelson, 1965).

**Figure 1:**
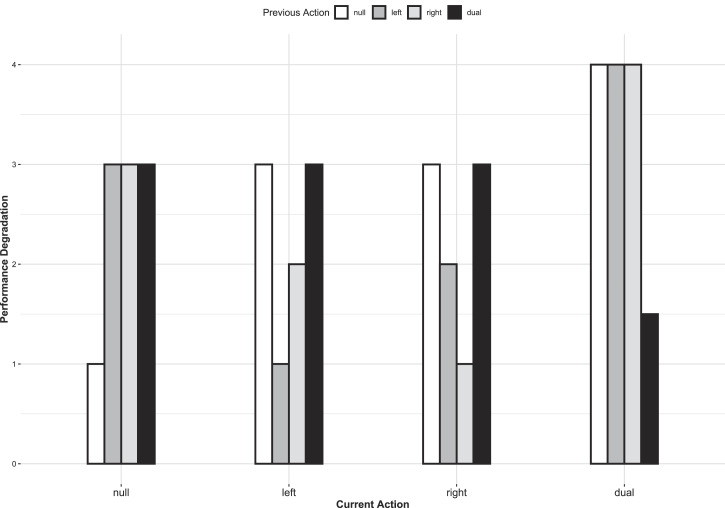
Idealized expected results in terms of performance degradation as a function of current action and previous action.

We followed up on the interaction of Current Action × Previous Action (when it was significant) by fixing the factor current action and conducting pairwise *t*-tests between all possible levels of the factor previous action (see Table 1). For example, there are four “flavors” of right-action trials: (1) right (current action) after right (previous action), (2) right after null, (3) right after dual, and (4) right after left, resulting in six comparisons: 1 versus 2, 1 versus 3, 1 versus 4, 2 versus 3, 2 versus 4, and 3 versus 4. As shown in Figure 1, we expected better performance for (1) (right after right) than for 2, 3, or 4 (right after null, dual, or left) due to general action-repetition benefits. More importantly though, as per Prediction 2, we expected that 4 (right after left) would be associated with better performance than 2 (right after null) and 3 (right after dual). Using comparison 4 versus 2 as an illustration, Table 1 shows that ERs were indeed 11.8% lower when performing a right action on the current trial after having performed a left action on the previous trial (previous action 1) than when having performed a null action on the previous trial (previous action 2).

**Table 1:** Post hoc *t*-tests for Current Action × Previous Action resolved by current action

Current	Previous 1	Previous 2	*t* (ERs)	*p*	Diff (%)	*t* (RTs)	*p*	Diff (ms)
Dual	Dual	Null	5.14	**<.001**	−21.3	8.86	**<.001**	−57.7
	Dual	Left	5.79	**<.001**	−21.4	14.98	**<.001**	−65.7
	Dual	Right	8.13	**<.001**	−23.2	11.22	**<.001**	−63.1
	Left	Right	0.56	= .581	−1.8	0.66	= .518	2.6
	Null	Left	0.01	= .993	0.0	1.27	= .216	−8.1
	Null	Right	0.64	= .526	−1.8	0.92	= .366	−5.5
Null	Null	Dual	2.69	**= .012**	−3.5	NA	NA	NA
	Null	Left	2.71	**= .012**	−5.5	NA	NA	NA
	Null	Right	3.95	**= .001**	−6.1	NA	NA	NA
	Left	Right	0.42	= .680	−0.6	NA	NA	NA
	Dual	Left	1.03	= .312	−2.0	NA	NA	NA
	Dual	Right	1.4	= .172	−2.6	NA	NA	NA
Right	Right	Dual	6.76	**<.001**	−20.6	9.73	**<.001**	−71.2
	Right	Left	6.74	**<.001**	−11.1	5.61	**<.001**	−39.9
	Right	Null	6.5	**<.001**	−22.9	9.91	**<.001**	−68.0
	Left	Dual	3.39	**= .002**	−9.5	5.03	**<.001**	−31.2
	Left	Null	4.28	**<.001**	−11.8	4.85	**<.001**	−28.1
	Dual	Null	0.77	= .449	−2.3	0.46	= .652	3.2
Left	Left	Dual	6.63	**<.001**	−24.9	11.31	**<.001**	−76.0
	Left	Right	4.23	**<.001**	−13.0	8.7	**<.001**	−50.9
	Left	Null	4.95	**<.001**	−19.2	9.46	**<.001**	−68.3
	Right	Dual	3.74	**= .001**	−11.9	4.57	**<.001**	−25.2
	Right	Null	1.99	= .057	−6.2	3.64	**= .001**	−17.4
	Dual	Null	1.82	= .081	5.7	1.26	= .219	7.7
*Note*. Diff = difference. For each current action, we computed mean ER/RT differences between all possible transitions from the previous trial (i.e., “current action after previous action 1” − “current action after previous action 2”). Thus, negative values always indicate better performance (lower RT/ER) for the current action after previous action 1. Bold print indicates statistical significance.								

RTs were only analyzed for correct trials (i.e., where the response corresponded to the action indicated by the signal), reducing the factor (current) action to three levels since responses in the null condition were – by definition – incorrect. Overall RTs for the dual-action condition were derived by averaging left- and right-hand RTs. We report ϵ (Greenhouse & Geisser, 1959) when sphericity is violated.

For ease of exposition, we also conducted a simplified sequence analysis focused on dual-action effects. Here, two new variables were created: “Action condition” was derived from current action by renaming the factor levels “left” and “right” to “single” (followed by re-averaging) and removing the factor level “null” (meaning that the only two remaining factor levels were “single” vs. “dual”), and “sequence” was derived from previous action and current action by coding all action repetitions as “repeat” and all nonrepetitions as “switch.” We did this to test for an interaction of action condition and sequence in the form of sequence-dependent DABs (for action switches) versus DACs (for action repetitions). As pointed out in the introduction, we did not predict DABs in the current paradigm – however, *if* there were even weak inhibition-based DABs in uni-modal settings, *this* would be the condition where we would expect to see them.

### Transparency and Openness

This study was pre-registered (https://doi.org/10.17605/OSF.IO/H7UEN) based on a pilot experiment with the same design and sample size. We report how we determined our sample size, all data exclusions (if any), all manipulations, and all measures in the study. All data (including the pilot) are available at https://osf.io/bx864/?view_only=2e83278dcfde4038a142f9030787abdf. Data were analyzed using R, version 4.0.5 (R Core Team, 2021), and the package ggplot2, version 3.3.5 (Wickham, 2016).

## Results

### Error Data

**Figure 2:**
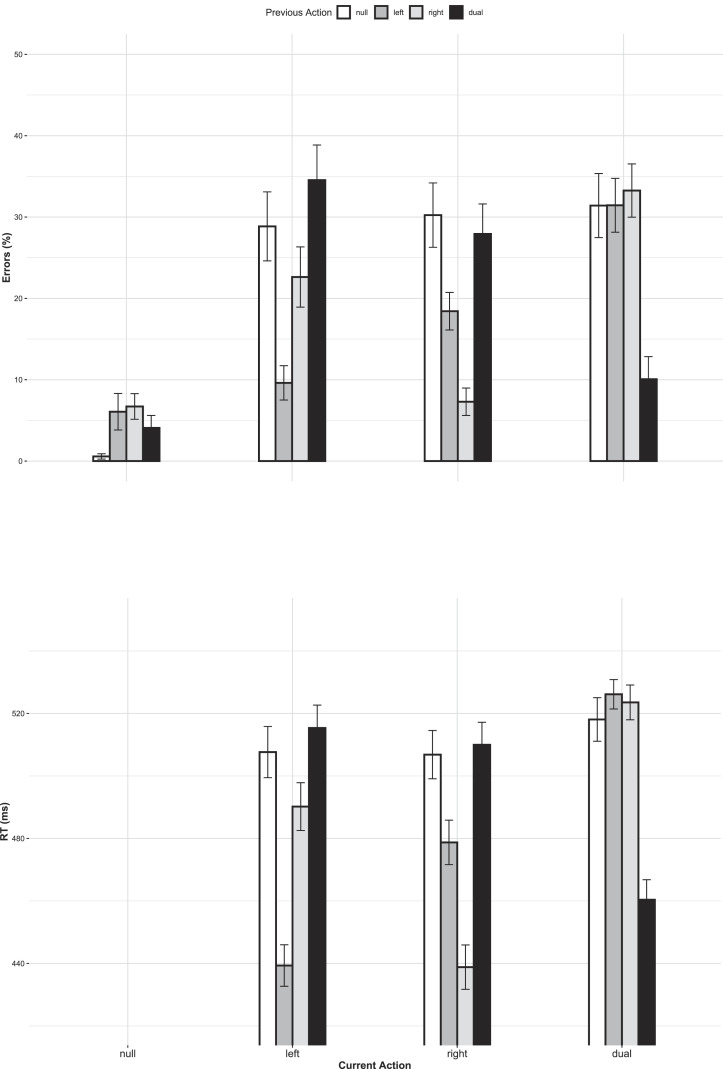
ERs (top panel) and RTs (bottom panel) as a function of current action and previous action for trials without mode instructions. *Note*. Error bars represent SE.

Regarding trial-by-trial sequence effects, both main effects [current action: F(3,78)=29.04, p<.001, ${\hat{\eta }}_{p}^{2}=.528$, ϵ=0.86, previous action: F(3,78)=7.75, p=.001, ${\hat{\eta }}_{p}^{2}=.230$, ϵ=0.74] as well as the interaction [F(9,234)=24.44, p<.001, ${\hat{\eta }}_{p}^{2}=.485$, ϵ=0.59; see Figure 2] were significant. Further resolving the interaction of Current Action × Previous Action revealed significant main effects of previous action on all levels of current action, indicating that for all four possible actions, performance was influenced by the action that had to be executed on the previous trial [current action = null: F(3,78)=5.11, p=.006, ${\hat{\eta }}_{p}^{2}=.164$, ϵ=0.78; left: F(3,78)=20.08, p<.001, ${\hat{\eta }}_{p}^{2}=.436$, ϵ=0.9; right: F(3,78)=26.73, p<.001, ${\hat{\eta }}_{p}^{2}=.507$, ϵ=0.74; dual: F(3,78)=20.58, p<.001, ${\hat{\eta }}_{p}^{2}=.442$, ϵ=0.85]. Follow-up pairwise *t*-tests (see Table 1) indicated strong ER repetition benefits for all actions. When current action = null or current action = dual, the remaining respective nonrepeating previous actions were not significantly different from each other, indicating that dual actions and null actions only profited from direct repetition. When current action = left or current action = right, on the other hand, nonrepetition performance was significantly better when the previous action had to be performed in the *opposite* direction (in contrast to a null action or dual actions) – thus, in terms of ERs, single-left and single-right actions not only profited from direct repetition, but also (to a lesser degree) from laterality switches, in line with Prediction 2 and in support of Hypothesis 2.

**Figure 3:**
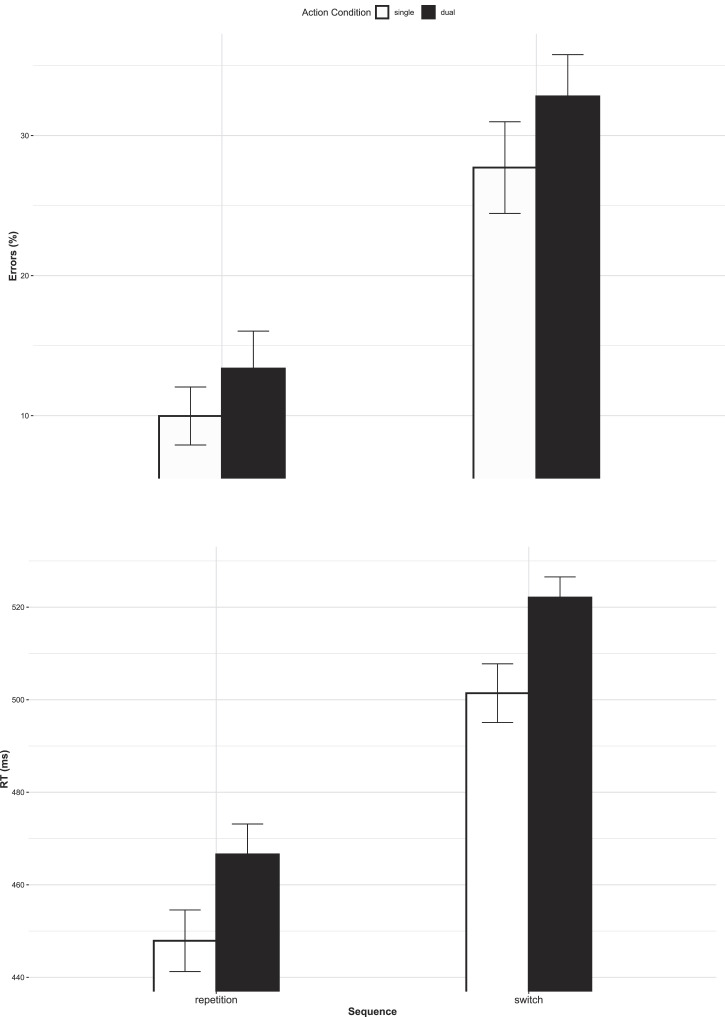
ERs (top panel) and RTs (bottom panel) as a function of action condition and sequence. *Note*. Error bars represent SE.

Regarding sequence-dependent dual-action effects (see Figure 3), both main effects were significant [sequence: F(1,26)=83.48, p<.001, ${\hat{\eta }}_{p}^{2}=.763$, repetition benefit 19%; action condition: F(1,26)=4.98, p=.034, ${\hat{\eta }}_{p}^{2}=.161$, DAC 4%], but the interaction was not [F(1,26)=0.46, p=.505, ${\hat{\eta }}_{p}^{2}=.017$]. Thus, even under ideal circumstances (i.e., in switch trials), there were no ER DABs. This prevalence of ER DACs – irrespective of sequence – is in line with Prediction 1 and in support of Hypothesis 1.

### Reaction Time Data

Overall, the RT results mirrored the ER results. Regarding trial-by-trial sequence effects, both main effects [current action: F(2,52)=13.40, p<.001, ${\hat{\eta }}_{p}^{2}=.340$, ϵ=0.96, previous action: F(3,78)=21.17, p<.001, ${\hat{\eta }}_{p}^{2}=.449$, ϵ=0.87] as well as the interaction [F(6,156)=86.45, p<.001, ${\hat{\eta }}_{p}^{2}=.769$, ϵ=0.64; see Figure 2] were significant. Further resolving the interaction of Current Action × Previous Action revealed significant main effects of previous action on all levels of current action, indicating that for all four possible actions, performance was influenced by the action that had to be executed on the previous trial [current action = left: F(3,78)=63.13, p<.001, ${\hat{\eta }}_{p}^{2}=.708$, ϵ=0.87; right: F(3,78)=48.08, p<.001, ${\hat{\eta }}_{p}^{2}=.649$, ϵ=0.95; dual: F(3,78)=63.59, p<.001, ${\hat{\eta }}_{p}^{2}=.710$, ϵ=0.8]. Follow-up pairwise *t*-tests (see Table 1) indicated strong RT repetition benefits for all actions. When current action = dual, the remaining respective nonrepeating previous actions were not significantly different from each other, indicating that dual actions and null actions only profited from direct repetition. When current action = left or current action = right, nonrepetition performance was significantly better when the previous action had to be performed in the *opposite* direction (in contrast to a null action or dual actions) – thus, in terms of RTs, single-left and single-right actions not only profited from direct repetition, but also (to a lesser degree) from laterality switches, in line with Prediction 2 and in support of Hypothesis 2.

Regarding sequence-dependent dual-action effects (see Figure 3), both main effects were significant [sequence: F(1,26)=218.71, p<.001, ${\hat{\eta }}_{p}^{2}=.894$, repetition benefit 55 ms; action condition: F(1,26)=25.51, p<.001, ${\hat{\eta }}_{p}^{2}=.495$, DAC 20 ms], but the interaction was not [F(1,26)=0.17, p=.683, ${\hat{\eta }}_{p}^{2}=.007$]. Thus, even under ideal circumstances (i.e., in switch trials), there were no RT DABs. This prevalence of RT DACs – irrespective of sequence – is in line with Prediction 1 and in support of Hypothesis 1.

## Discussion

In the present paper, we investigated the effects of single- versus dual-action demands in a simple, uni-modal, single-onset paradigm. As predicted (Prediction 1), we did not observe DABs, but rather significant DACs, in line with the assumption that DABs are based on differential inhibitory costs due to inhibitory coding (Raettig & Huestegge, 2021, 2023) and in line with Hypothesis 1: Inhibitory coding is only employed in contexts where inhibition is easy and cost-effective (i.e., when multi-modal responses reduce the potential for intra-modal interference and crosstalk). Furthermore, in line with Prediction 2, directional switches were associated with better performance than all other kinds of switches, supporting Hypothesis 2: actions appear to be coded as bundles of distinctive features (entailing that left-to-right and right-to-left switches simply require inverting the polarity of a “laterality” feature).

### Uni- Versus Multi-Modal Inhibitory Costs

In comparison to executive dual-action representations (“execute A + execute B”), inhibitory dual-action representations are less resource demanding since they allow for noncompositional coding (“inhibit nothing” when dual-action execution is a highly automatized default behavior, Raettig & Huestegge, 2021, 2023). Inhibition as a *process*, though, can still be quite costly, and the data reported here are compatible with the interpretation that under uni-modal response demands, single-action inhibitory costs (“inhibit A” to execute B, again assuming a dual-action default) become so high that inhibitory coding is no longer beneficial, leading to executive coding instead. This effect can be explained by a high potential for crosstalk in the form of intra-modal response-code conflict (see Paas Oliveros et al., 2023; Schacherer & Hazeltine, 2021; Schuch & Koch, 2004): *inhibiting* response A interferes with *executing* response B if both responses pertain to the same modality (e.g., it is hard to respond with the left hand while at the same time *not* responding with the right hand). Multi-modal inhibitory coding, on the other hand, does not come with a comparable crosstalk penalty, indicating that inhibitory codes do not (as easily) spread between effector systems, possibly due to some form of weak encapsulation (Fodor, 1983).

The above argument entails that intra-modal response-code conflict is more costly than cross-modal response-code conflict, at least when the response codes in question are inhibitory (vs. executive). However, a number of previous studies have found seemingly conflicting patterns of results, albeit using notably different experimental paradigms (Huestegge et al., 2014; Weller et al., 2022). Weller et al. (2022) had participants respond to a single-onset auditory stimulus by pressing a button, executing a saccade, or both. Crucially, multi-modal dual actions (i.e., button press and saccade) were *intermixed* with uni-modal (manual) dual actions (i.e., left-hand button press and right-hand button press). The results indicated DACs, *not* DABs, for *both* uni-modal and multi-modal dual actions – thus, it seems that inhibitory coding was prevented because either (1) it is impossible to derive a useful (dual-action) default given *two different* dual-action conditions or (2) uni-modal inhibitory crosstalk in *some* conditions (i.e., single manual) is sufficient to make inhibitory coding globally unviable.

Huestegge et al. (2014) used visual and auditory stimuli triggering a vocal response, a saccade, or both at the same time. In contrast to Weller et al. (2022), there was no uni-modal dual-action condition here. Nevertheless, the results indicated clear DACs for both modalities, again indicating an absence of inhibitory coding in a multi-modal setting. However, conditions were presented in a *blocked* fashion, resulting in a reductive response set (Raettig & Huestegge, 2023): In each single-action block, the alternative single action was never required (e.g., participants knew that in the single-manual block, they would never have to execute a saccade), making it highly unlikely that the alternative response would have to be actively suppressed (put differently: a dual-action default would be very inefficient in a single-action block).

In sum, the current results suggest that multi-modality is a necessary, but not a sufficient condition for inhibitory coding. Looking at the literature, other preconditions appear to be a single, multi-modal dual-action condition (cf. Weller et al., 2022) as well as a nonreductive response set (cf. Huestegge et al., 2014; Raettig & Huestegge, 2023). Future research could investigate these factors in more detail; furthermore, it would be interesting to *directly* contrast uni-modal versus multi-modal dual actions using the same basic paradigm (similar to Weller et al., 2022, but using a between-subject design to allow for inhibitory coding in the multi-modal group).

### Inverting Binary Action Features

While performance always suffered when action demands changed from trial to trial, a striking result of the sequential analysis was that alternating between both single-action responses was associated with significantly better performance than all other types of action switches. This result can be explained parsimoniously when action plans are conceptualized as specifications of distinctive features (Rosenbaum, 1980; also see Chomsky, 1965; Jakobson & Halle, 1956) – for example, effector (“index finger”), laterality (“left”), and movement (“down”). Crucially, switching a single, *binary* feature specification to its *opposite* should be relatively easy, especially in contrast to more complex operations involving more fundamental changes to the previous response configuration (e.g., creating a full feature specification from scratch when the previous trial was a null trial, but the current trial is a single-action trial).

The question of how actions are cognitively represented can be approached from different perspectives. We have proposed that on a very general level, executive versus inhibitory coding determines if an action representation encodes what to *do* or what *not* to do (Raettig & Huestegge, 2021, 2023). Specific actions themselves (i.e., the “what”) are then coded on the basis of distinctive features. This concept of action representations as feature bundles (Rosenbaum, 1980) is a cornerstone of prominent current theories of action control (Frings et al., 2020). Our results thus generally support these frameworks, although it should be noted that explaining our particular observations does not require the inclusion of perceptual features or action effects into the action representation (as is typical in that branch of theories).

Regarding the cognitive processes that operate on these action representations, we have proposed that at least actions with clearly defined polar opposites (in a given context) can be “inverted” at a relatively low cost. This idea has some antecedents in the literature (e.g., Adam et al., 2014; Hedge & Marsh, 1975), but our experimental paradigm notably differs from the corresponding studies: In both the anti-cue paradigm (Adam et al., 2014) and the reverse Simon procedure (Hedge & Marsh, 1975), participants have to do the opposite of what a particular stimulus *on the current trial* suggests. In the present experiment, on the other hand, it is not the meaning of the *stimulus* that has to be inverted, but a singular feature of the action executed on the previous trial. This action modification then results in a new action plan which – when executed – produces a correct response (i.e., in line with the current, noninverted stimulus). Importantly, “surgical” action modification as envisioned here is much more efficient than deactivating the old action in its entirety and restarting the response selection process from scratch (the latter being similar to strategies that have been observed in the stop-signal literature, e.g., “Stop-then-discriminate”; cf. Bissett & Logan, 2014). Nevertheless, similar to inhibitory coding, action modification – although generally more efficient – may not always be possible. Future research could investigate this (i.e., action modification vs. resetting and reselecting) in more detail.

### Conclusions

In conclusion, the pattern of results reported here strengthens the case for multi-modality (in the responses) as a critical prerequisite for inhibition-based dual-action benefits (in the RTs). It appears that concurrent intra-modal action inhibition and execution (e.g., executing a left button press while inhibiting a right button press) is too inefficient to warrant inhibitory coding. Moreover, our results are in line with the assumption that actions are represented as feature bundles. Interestingly, it appears that at least actions with polar distinctive features (e.g., laterality) can be modified by a relatively inexpensive inversion operation to quickly adapt a previous response to new, changed action demands. More generally speaking, this implies that action modification can make partial (beneficial) use of preconfigured action representations whenever the generic action category (e.g., “button press”) is repeated, but only its concrete parametrization (e.g., left vs. right) needs to be adjusted.

## Electronic Supplementary Materials

The electronic supplementary materials are available with the online version of the article at https://doi.org/10.1027/1618-3169/a000604

**ESM 1.** Illustration of procedure.

**ESM 2.** Analysis of mode.

## Footnotes

^1^
Note that all traditional theories of multiple-action control would predict DACs for executively coded dual actions (e.g., due to a response-selection bottleneck).

^2^
An illustration of the experimental procedure is supplied as the Electronic Supplementary Material, ESM 1.

## Notes

**Authorship:** The authors made the following contributions. Tim Raettig: Conceptualization, Investigation, Formal Analysis, Writing – Original Draft Preparation, Writing – Review & Editing; Lynn Huestegge: Supervision, Writing – Review & Editing.

**Open Science:** This study was pre-registered (https://doi.org/10.17605/OSF.IO/H7UEN) based on a pilot experiment with the same design and sample size. We report how we determined our sample size, all data exclusions (if any), all manipulations, and all measures in the study. All data (including the pilot) are available at https://osf.io/bx864/?view_only=2e83278dcfde4038a142f9030787abdf (Raettig, 2024).

## References

[c1] Adam, J., Bovend’Eerdt, T., Smulders, F., & Van Gerven, P. (2014). Strategic flexibility in response preparation: Effects of cue validity on reaction time and pupil dilation. *Journal of Cognitive Psychology*, *26*(2), 166–177. 10.1080/20445911.2014.883399

[c2] Bertelson, P. (1965). Serial choice reaction-time as a function of response versus signal-and-response repetition. *Nature*, *206*(980), 217–218. 10.1038/206217a0 5830165

[c3] Bissett, P. G., & Logan, G. D. (2014). Selective stopping? Maybe not. *Journal of Experimental Psychology: General*, *143*(1), 455–472. 10.1037/a0032122 23477668 PMC3728275

[c4] Chomsky, N. (1965). *Aspects of the theory of syntax* (50th ed.). The MIT Press. https://www.jstor.org/stable/j.ctt17kk81z

[c5] Fagot, C., & Pashler, H. (1992). Making two responses to a single object: Implications for the central attentional bottleneck. *Journal of Experimental Psychology: Human Perception and Performance*, *18*(4), 1058–1079. 10.1037/0096-1523.18.4.1058 1431744

[c6] Fodor, J. A. (1983). *Modularity of mind*. MIT Press.

[c7] Frings, C., Hommel, B., Koch, I., Rothermund, K., Dignath, D., Giesen, C., Kiesel, A., Kunde, W., Mayr, S., Moeller, B., Möller, M., Pfister, R., & Philipp, A. (2020). Binding and retrieval in action control (BRAC). *Trends in Cognitive Sciences*, *24*(5), 375–387. 10.1016/j.tics.2020.02.004 32298623

[c8] Greenhouse, S. W., & Geisser, S. (1959). On methods in the analysis of profile data. *Psychometrika*, *24*(2), 95–112. 10.1007/BF02289823

[c9] Hedge, A., & Marsh, N. W. A. (1975). The effect of irrelevant spatial correspondences on two-choice response-time. *Acta Psychologica*, *39*(6), 427–439. 10.1016/0001-6918(75)90041-4 1199779

[c10] Huestegge, L. (2011). The role of saccades in multitasking: Towards an output-related view of eye movements. *Psychological Research*, *75*(6), 452–465. 10.1007/s00426-011-0352-5 21720887

[c11] Huestegge, L., & Koch, I. (2014). When two actions are easier than one: How inhibitory control demands affect response processing. *Acta Psychologica*, *151*, 230–236. 10.1016/j.actpsy.2014.07.001 25086224

[c12] Huestegge, L., Pieczykolan, A., & Koch, I. (2014). Talking while looking: On the encapsulation of output system representations. *Cognitive Psychology*, *73*, 72–91. 10.1016/j.cogpsych.2014.06.001 25003309

[c13] Huestegge, L., & Strobach, T. (2021). Structuralist mental representation of dual-action demands: Evidence for compositional coding from dual tasks with low cross-task dimensional overlap. *Acta Psychologica*, *216*, Article 103298. 10.1016/j.actpsy.2021.103298 33774503

[c14] Jakobson, R., & Halle, M. (1956). *Fundamentals of language*. Mouton & Co.

[c15] Kürten, J., Raettig, T., Gutzeit, J., & Huestegge, L. (2022). Dual-action benefits: Global (action-inherent) and local (transient) sources of action prepotency underlying inhibition failures in multiple action control. *Psychological Research*, *87*(2), 410–424. 10.1007/s00426-022-01672-0 35394557 PMC9928916

[c16] Kürten, J., Raettig, T., Gutzeit, J., & Huestegge, L. (2023). Preparing for simultaneous action and inaction: Temporal dynamics and target levels of inhibitory control. *Journal of Experimental Psychology: Human Perception and Performance*, *49*(7), 1068–1082. 10.1037/xhp0001126 37227859

[c17] Lakens, D., & Caldwell, A. R. (2021). Simulation-based power analysis for factorial analysis of variance designs. *Advances in Methods and Practices in Psychological Science*, *4*(1), 1–14. 10.1177/2515245920951503

[c18] Meyer, D. E., & Kieras, D. E. (1997). A computational theory of executive cognitive processes and multiple-task performance: Part I. Basic mechanisms. *Psychological Review*, *104*(1), 3–65. 10.1037/0033-295X.104.1.3 9009880

[c19] Navon, D., & Miller, J. (1987). Role of outcome conflict in dual-task interference. *Journal of Experimental Psychology: Human Perception and Performance*, *13*(3), 435–448. 10.1037/0096-1523.13.3.435 2958592

[c20] Paas Oliveros, L. K., Pieczykolan, A., Pläschke, R. N., Eickhoff, S. B., & Langner, R. (2023). Response-code conflict in dual-task interference and its modulation by age. *Psychological Research*, *87*(1), 260–280. 10.1007/s00426-021-01639-7 35122495 PMC9352817

[c21] Pashler, H. (1994). Dual-task interference in simple tasks: Data and theory. *Psychological Bulletin*, *116*(2), 220–244. 10.1037/0033-2909.116.2.220 7972591

[c22] R Core Team (2021). *R: A language and environment for statistical computing*. Manual. R Foundation for Statistical Computing. https://www.R-project.org/

[c23] Raettig, T. (2024). *Dual-action costs and benefits in a uni-modal single-onset paradigm* [Data]. https://osf.io/bx864/?view_only=2e83278dcfde4038a142f9030787abdf 10.1027/1618-3169/a000604 PMC12529621 38602117

[c24] Raettig, T., & Huestegge, L. (2018). The hard work of doing nothing: Accounting for inhibitory costs during multiple action control. *Attention, Perception, & Psychophysics*, *80*(7), 1660–1666. 10.3758/s13414-018-1577-9 30069681

[c25] Raettig, T., & Huestegge, L. (2021). Representing action in terms of what not to do: Evidence for inhibitory coding during multiple action control. *Journal of Experimental Psychology: Human Perception and Performance*, *47*(9), 1253–1273. 10.1037/xhp0000943 34694854

[c26] Raettig, T., & Huestegge, L. (2023). Explaining dual-action benefits: Inhibitory control and redundancy gains as complementary mechanisms. *Journal of Experimental Psychology, Learning, Memory, and Cognition*. Advance online publication. 10.1037/xlm0001231 37079846

[c27] Rosenbaum, D. A. (1980). Human movement initiation: Specification of arm, direction, and extent. *Journal of Experimental Psychology: General*, *109*(4), 444–474. 10.1037/0096-3445.109.4.444 6449531

[c28] Schacherer, J., & Hazeltine, E. (2021). Crosstalk, not resource competition, as a source of dual-task costs: Evidence from manipulating stimulus-action effect conceptual compatibility. *Psychonomic Bulletin & Review*, *28*(4), 1224–1232. 10.3758/s13423-021-01903-2 33689145

[c29] Schuch, S., & Koch, I. (2004). The Costs of changing the representation of action: Response repetition and response–response compatibility in dual tasks. *Journal of Experimental Psychology: Human Perception and Performance*, *30*(3), 566–582. 10.1037/0096-1523.30.3.566 15161387

[c30] Tombu, M., & Jolicoeur, P. (2003). A central capacity sharing model of dual-task performance. *Journal of Experimental Psychology, Human Perception and Performance*, *29*(1), 3–18. 10.1037/0096-1523.29.1.3 12669744

[c31] Weller, L., Pieczykolan, A., & Huestegge, L. (2022). Response modalities and the cognitive architecture underlying action control: Intra-modal trumps cross-modal action coordination. *Cognition*, *225*, Article 105115. 10.1016/j.cognition.2022.105115 35390694

[c32] Wickens, C. D. (2008). Multiple resources and mental workload. *Human Factors: The Journal of the Human Factors and Ergonomics Society*, *50*(3), 449–455. 10.1518/001872008X288394 18689052

[c33] Wickham, H. (2016). *Ggplot2: Elegant graphics for data analysis*. Springer-Verlag. https://ggplot2.tidyverse.org

