# Supplementary material for: Dual-Action Costs and Benefits in a Uni-Modal Single-Onset Paradigm
Source: Exp Psychol. 2024 Apr 11;70(6):344–54. doi: 10.1027/1618-3169/a000604 (PMC12529621; doi:10.1027/1618-3169/a000604)
Supplement: Supplementary file 1 [file zea_70_6_344_esm2.pdf]

Supplementary Materials

Data Analysis. We report the analysis of ERs and RTs as a function of the within-subject independent variables signal (null, left, right, dual) and mode ("Think of [x]" = think_x, "Do not think of [x]" = suppress_x, "Think of anything" = free).

RTs were only analyzed for correct trials (i.e., where the response corresponded to the signal), reducing the factor (current) signal to three levels since responses in the null condition were – by definition – incorrect. We report corrected degrees of freedom (Greenhouse & Geisser, 1959) where appropriate.

Results

Error Data. The main effect of signal ($F(2.62, 68.15) = 31.35$, $p < .001$, $\hat{\eta}^2_p = .547$) was significant, but the main effect of mode ($F(1.87, 48.63) = 0.58$, $p = .555$, $\hat{\eta}^2_p = .022$) and the interaction of Signal×Mode ($F(4.59, 119.36) = 1.52$, $p = .194$, $\hat{\eta}^2_p = .055$; see Figure 1) were not.

Reaction Time Data. The main effect of signal ($F(1.95, 50.70) = 11.74$, $p < .001$, $\hat{\eta}^2_p = .311$) was significant, but the main effect of mode ($F(1.84, 47.82) = 0.86$, $p = .423$, $\hat{\eta}^2_p = .032$) and the interaction of Signal×Mode ($F(3.08, 80.06) = 1.27$, $p = .289$, $\hat{\eta}^2_p = .047$; see Figure 1) were not.

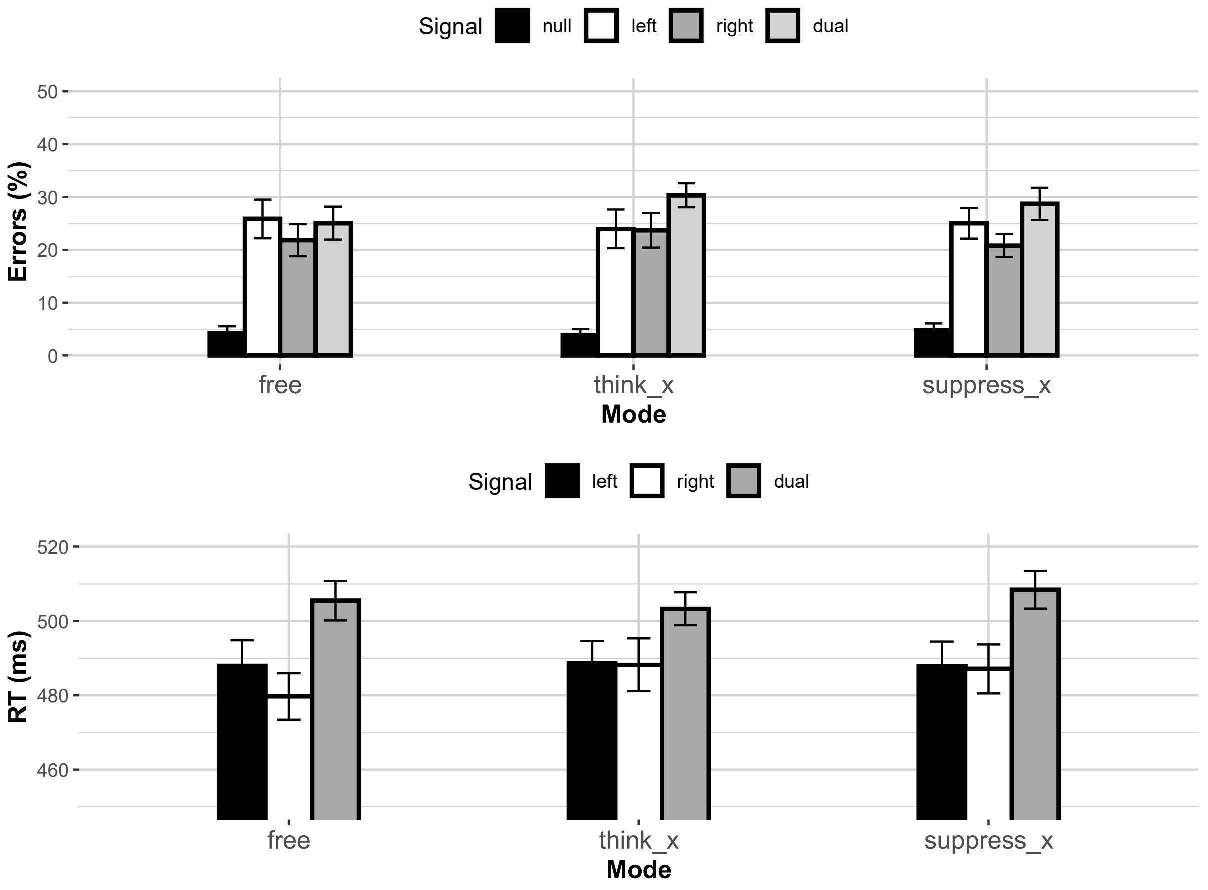

Figure 1. ERs (top panel) and RTs (bottom panel) as a function of current signal and mode. Error bars represent SE.
