# Supplementary figures and images for: Dual-Action Costs and Benefits in a Uni-Modal Single-Onset Paradigm
Source: Exp Psychol. 2024 Apr 11;70(6):344–54. doi: 10.1027/1618-3169/a000604 (PMC12529621; doi:10.1027/1618-3169/a000604)

Signal

Every 4th trial

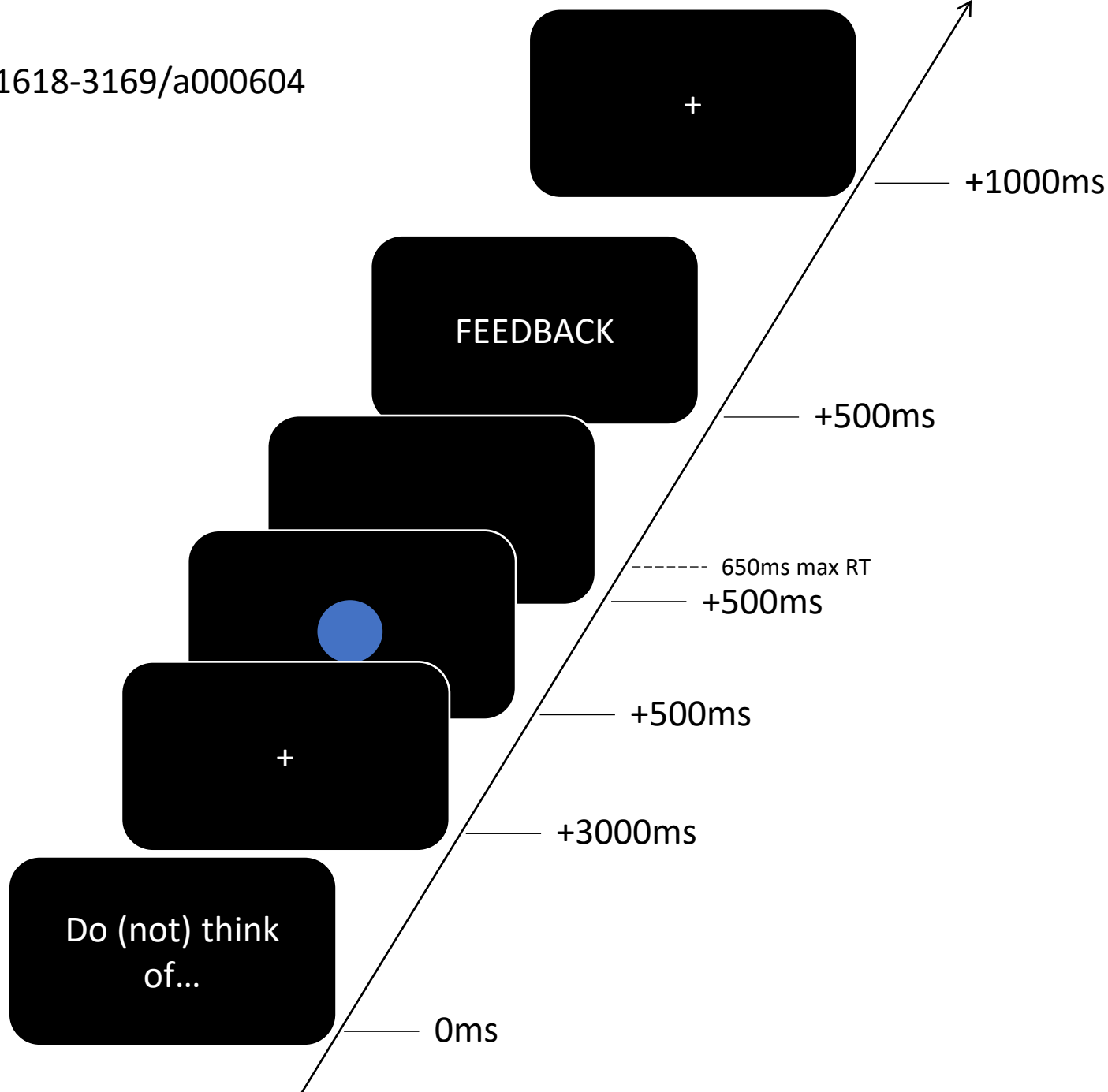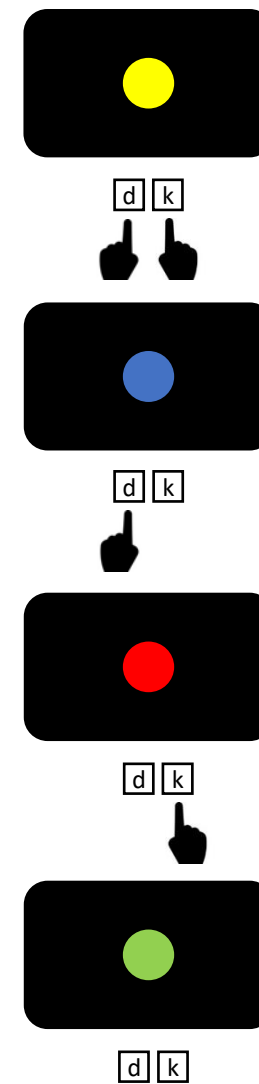

Supplement: Supplementary file 2 [file zea_70_6_344_esm1.pdf]
